# Structural Characterization of Polysaccharide from *Flammulina velutipes* and Its Impact on Hyperlipidemia Through Modulation of Hepatic Cholesterol Metabolism and Gut Microbiota

**DOI:** 10.3390/foods14193452

**Published:** 2025-10-09

**Authors:** Wei Jia, Huimin Wang, Ting Feng, Xiaoxiao Liu, Zhendong Liu, Zhengpeng Li, Wenhan Wang, Jingsong Zhang

**Affiliations:** 1 Institute of Edible Fungi, Shanghai Academy of Agricultural Sciences, Shanghai 201403, China; jiawei@saas.sh.cn (W.J.);; 2 College of Food Science and Technology, Shanghai Ocean University, Shanghai 201306, China; 3 Food Science College, Tibet Agriculture & Animal Husbandry University, Nyingchi 860000, China

**Keywords:** *Flammulina velutipes*, polysaccharide, structure, hyperlipidaemia, lipid-lowering effect

## Abstract

FVPB1, a novel heteropolysaccharide, was extracted from the *Flammulina velutipes* fruiting body, and its structure was determined by methylation analysis, nuclear magnetic resonance (NMR) spectroscopy. FVPB1 demonstrated efficacy in inhibiting lipid accumulation in Raw264.7 cells and zebrafish, as well as in reducing weight gain and ameliorating liver injury in high-fat diet-induced mice. High concentration of FVPB1 significantly increased serum ApoA1 levels, while all tested doses (low, medium, and high) reduced serum ApoB levels in mice. Intervention with FVPB1 significantly increased the mRNA expression of Lcat and Cyp7a1 enzymes while markedly reducing the transcriptional level of Hmgcr reductase. Additionally, low concentration of FVPB1 enhanced CYP7A1 protein expression, whereas medium and high concentrations of FVPB1 promoted LCAT protein expression. Medium and high concentrations of FVPB1 significantly enhanced bile acid excretion in mice, with the high dose additionally promoting fecal sterol output. Alpha and beta diversity analyses demonstrated that a high-fat diet induced substantial dysbiosis in the gut microbiota of mice, characterized by reduced microbial diversity and richness. Intervention with FVPB1 significantly modulated the structural composition of the intestinal microbiota in high-fat diet-fed mice. Therefore, FVPB1 exerts lipid-lowering effect in high-fat diet-fed mice by modulating cholesterol metabolism and ameliorating gut microbiota dysbiosis.

## 1. Introduction

Hyperlipidemia (or Hyperlipidaemia), the most prevalent metabolic disorder worldwide, is characterized by abnormal lipid levels. Characterized by high blood lipid levels due to metabolic changes, such as increased triglycerides (TGs), total cholesterol, or low-density lipoprotein cholesterol (LDL-C), or decreased high-density lipoprotein cholesterol (HDL-C) [1]. It is classified into primary (genetic) and secondary (acquired) types.

Primary hyperlipidemia is caused by mutations in proteins involved in lipid and lipoprotein metabolic pathways [2]. In contrast, secondary hyperlipidemia may develop due to overnutrition, physical inactivity, and excessive alcohol consumption. Considering its close relationship with various cardiovascular diseases, reducing blood lipid levels is an important strategy for reducing cardiovascular risk and improving overall societal health [3]. Lifestyle interventions, including dietary changes, establishing consistent routines, engaging in appropriate physical activity, and exercising, may help regulate blood lipid concentrations. However, these non-pharmacological methods are limited by delayed efficacy and challenges associated with long-term maintenance. Therefore, pharmacological treatments, particularly statins, fibrates, niacin and its derivatives, bile acid chelators, and ezetimibe, are commonly used to manage blood lipid levels. However, extended use may cause toxic side effects, such as kidney disease, myopathy, liver injury, and gastrointestinal diseases. Hence, research is ongoing into the efficacy and safety of natural substances with low toxicity and lipid-lowering activities.

Edible fungi are rich in proteins, amino acids [4], and various physiologically active substances, including polysaccharides [5], terpenoids [6], sterols [7], alkaloids [8], and glycosides [9], offering antibacterial, antitumour, and blood pressure-lowering benefits [10]. Specifically, the polysaccharides isolated from fruiting bodies, and mycelia, exert antioxidant [11], antitumour [12], antiviral [13], antibacterial [14], anti-inflammatory [15], immune regulatory [16], blood sugar lowering [17], and blood lipid lowering [18] properties.

Mechanistically, polysaccharides from edible fungi attenuate dyslipidemia through modulating cholesterol metabolism [19,20], enhancing lipid catabolism [19,20], and regulating gut microbiota [19,20,21], and oxidative stress [21,22]. For example, *Pleurotus eryngii* polysaccharides regulate total cholesterol (TC), TG, LDL-C, and very LDL-C (VLDL-C) levels, decrease HDL-C in STZ-induced diabetic mice [23], alter the intestinal microbial community structure in high-fat model mice, and increase bile acid secretion and lipid excretion [24]. *Cordyceps* polysaccharides help reduce blood and liver lipid levels and restore lipid metabolism disorders caused by high-fat emulsions [25]. Additionally, *C. militaris* polysaccharides can reverse gut microbiota dysbiosis caused by a high-fat diet and improve metabolite profiles, indicating potential as prebiotics [26]. *Ganoderma lucidum* extract inhibits cholesterol synthesis in T9A4 liver cells in vitro [27]. Similarly, in vivo, it decreases serum TC and LDL-C levels as well as liver HMG CoA reductase (HMGCR) activity in hamsters, partly by increasing fecal sterol, total bile acid, and chenodeoxycholic acid contents, suggesting its role in regulating cholesterol synthesis and absorption.

*Flammulina velutipes* (Curtis) Singer—also known as winter mushroom, white mushroom, intellectual mushroom, silk mushroom, hairy stem money mushroom, and “Yixiu mushroom” and “Zhizhi mushroom” in Japan—is rich in zinc, supporting children’s growth and cognition. The polysaccharides of *F. velutipes* fruiting body exert potential antitumour, immune-regulatory, antiviral, lipid-lowering, anti-fatigue, and liver-protective effects [28].

In a preliminary study, we isolated, purified, and structurally identified five polysaccharides from *F. velutipes*: FVPA1, FVPA2, FVPB1, FVPB2, and FVPT1. FVPA2 stimulates Raw264.7 macrophages to secrete Nitric Oxide (NO) [28]. FVPA1 significantly increases natural killer cell activity against K562 target cells in a dose-dependent manner [29]. FVPB2 induces mouse splenic lymphocyte proliferation in a dose-dependent manner and increases IgM and IgG secretion levels by B cells [30]. FVPT1 exhibits marked immune regulatory activity by increasing NO, interleukin (IL)-1β, and IL-1 secretion by macrophages.

Previous studies have demonstrated that crude polysaccharides from *F. velutipes* significantly reduce serum levels of TC, TG, and LDL-C, while increasing HDL-C levels and ameliorating hepatic steatosis in obese mice [31]. These lipid-lowering effects are achieved through the promotion of lipid oxidation and inhibition of lipid synthesis [31]. However, in contrast to these previous studies, FVPB1 is a purified and structurally homogeneous polysaccharide derived from the crude extracts of *F. velutipes*. To date, the lipid-lowering effect and underlying mechanisms of FVPB1 have not been investigated. The present study aims to examine the regulatory effects of FVPB1 on hepatic cholesterol metabolism and gut microbiota composition in mice, thereby providing a theoretical foundation for further exploration of the lipid-lowering mechanisms of *F. velutipes* polysaccharides.

## 2. Materials and Methods

### 2.1. Materials

The fruiting bodies of *F. velutipes* were provided by Jiangsu Hualv Biotechnology Co. Ltd. (Suqian, China). Sephacryl S-300 High Resolution (XK16 mm × 100 cm, 50 μm) and DEAE Sepharose Fast Flow (XK26, ×100 cm, 90 μm) were purchased from GE Healthcare (GE Healthcare, Cardiff, UK). Trifluoroacetic acid (TFA), and monosaccharide standards (Fucose (D-Fuc), Rhamnose (L-Rha), Arabinose (D-Ara), Glucosamine (GA), Galactose (D-Gal), Glucose (D-Glc), Xylose (D-Xyl), Mannose (D-Man), Fructose (L-Fruc), Gluconic acid (GalA) and galacturonic acid (GalA)) were purchased from Sigma-Aldrich (St. Louis, MI, USA). All other reagents were of analytical grade and sourced from the China National Pharmaceutical Group.

### 2.2. FVPB1 Separation and Purification

The fruiting bodies of *F. Velutipes* (1.0 kg), defatted with 95% EtOH for 12 h, were extracted with boiling water for two times, 2 h for each time. The liquid extracts were combined, centrifuged (26,000× *g*, 20 min, 20 °C), and concentrated to one-tenth of the original volume. 95% EtOH was added until the final alcohol concentration reached 30% for precipitation. The precipitated was separated out and lyophilized as FVP30 (8.6 g, yield 0.86%). Ethanol (95%) was subsequently added until the final alcohol concentration reached 60% and lyophilized as FVP60 (11.2 g, yield 1.12%). An aliquot (10 g) of FVP60 was resuspended in 100 mL distilled water and centrifuged as before. The supernatant was applied to a DEAE-Sepharose Fast Flow column, eluted first with distilled water and then with a 0 to 2 M gradient of NaCl. The fractions were collected and the carbohydrate fraction was detected with a phenol–sulfuric acid method [32]. FVP60A (4.5 g, yield 0.45%) was obtained from the water eluate and FVP60B (2 g, yield 0.22%) was obtained from the 0 to 2 M gradient NaCl eluate from FVP60 [28]. FVP60B was dialyzed (cut-off 8.0–10 kDa) for 4 days against 20 changes in distilled water, concentrated to ∼10 mL under reduced pressure at 40 °C and lyophilized, dissolved in distilled water to 10 mg/mL, centrifuged at 7000× *g* for 5 min at room temperature, and 2 mL of supernatant was placed on a Sephacryl S-300 column using distilled water as the mobile phase at 1 mL/min. Fractions were collected at 1.2× column volume detected on differential refractive index detector (RI) (RID-10A, Shimadzu Corporation, Kyoto, Japan). The first peak was collected and freeze-dried to yield FVPB1 (0.6 g, yield 0.07%).

### 2.3. Polysaccharide and Protein Content Determination

Polysaccharide content was determined using the phenol–sulfuric acid method [32]. The protein concentration was determined using a bicinchoninic acid (BCA) Protein Assay Kit (Thermo Fisher Scientific, Waltham, MA, USA) [33].

### 2.4. Infrared Spectroscopy Analysis

FVPB1 (2 mg) was ground with KBr, uniformly blended, and compressed into tablets. Infrared spectroscopy scanning was conducted within the 4000–400 cm^−1^ range.

### 2.5. Determination of Purity and Molecular Weight

Homogeneity and the molecular weight of FVPB1 were determined by High Performance Liquid Chromatography (HPLC) using a Waters 2695 HPLC system equipped (Waters Corporation, Milford, MA, USA) with refractive index detector (RI) and a UV detector for concentration determination, fitted with TSK PWXL 6000 and 3000 gel filtration columns. Aliquots (100 μL) of FVPB1 solution (2 mg/mL buffer consisting of 0.15 M NaNO_3_ and 0.05 M NaH_2_PO_4_, pH 7) were applied to the column and eluted with the same buffer at a flow rate of 0.5 mL/min. The column was calibrated using pullulan standards, P5 (6200 Da), P10 (10,000 Da), P20 (21,700 Da), P100 (113,000 Da), P200 (200,000 Da) (Showa Denko, Tokyo, Japan). The column temperature and RI detector temperature were maintained at 35 °C [34].

### 2.6. Monosaccharide Composition Analysis

Samples (2 mg) were hydrolyzed with 2 M trifluoroacetic acid (TFA) at 110 °C for 4 h, and the resulting monosaccharides were identified by high-performance anion exchange pulsed-amperometric detection chromatography (HPAEC-PAD) using a Dionex LC30 instrument equipped with a CarboPacTM PA20 column (3 mm × 150 mm) (Thermo Fisher Scientific Inc., Waltham, MA, USA). The column was eluted with the mobile phases A, B and C (consisting of deionized water, 250 mmol/L NaOH and 1 mol/L NaAc, respectively) used in the following combinations (A, B, C): 0–30 min, (99.2:0.8:0), 30–40 min (99.2:0.8:0–79.2:0.8:20), 40–40.1 min (79.2:0.8:20–20:80:0), and 40.1–60 min (20:80:0–99.2:0.8:0). The sample volume, column temperature and flow rate were 25 µL, 30 °C and 0.45 mL/min, respectively. Monosaccharide components and percentage composition were determined using monosaccharide standards [30].

### 2.7. Methylation Analysis

Vacuum-dried FVPB1 (2 mg) was dissolved in Dimethyl sulfoxide (DMSO) (1 mL) and methylated with a solution of 2.5% NaOH in DMSO (1 mL) and CH_3_I (0.5 mL) for 30 min at room temperature. The reaction mixture was extracted with 0.5 mL CHCl_3_, the organic phase was washed 3× with 2–3 mL water, and the solvent was then removed by evaporation under reduced pressure. Complete methylation was confirmed by the disappearance of the -OH band (3200–3700 cm^−1^) in FTIR spectrum. Permethylated polysaccharide was hydrolyzed with HCOOH (88%, 3 mL) at 100 °C for 3 h, evaporated to dryness and further hydrolyzed with 2 M TFA (4 mL) at 110 °C for 4 h. The partially methylated oligosaccharide in the hydrolyzate was reduced with NaBH_4_ and acetylated with Ac_2_O, and the resulting mixture of methylated alditol acetates was analyzed by GC–MS using a DB-5MS column (30 m × 0.25 mm × 0.25 mm) and a temperature program consisting of 180–270 °C at 20 °C/min, and held at 270 °C for 25 min [30].

### 2.8. Nuclear Magnetic Resonance (NMR)

The FVPB1 was dissolved with D_2_O and lyophilized in a vacuum freeze dryer to facilitate deuterium exchange. The deuterium-exchanged FVPB1 (35 mg) was dissolved in 0.5 mL of 99.96% D_2_O for NMR. ^1^H,^13^C, Nuclear Overhauser Effect Spectroscopy (NOESY), ^1^H-^1^H-correlated spectroscopy (COSY), heteronuclear multiple quantum coherence (HMQC) and ^1^H-detected heteronuclear multiple-bond correlation (HMBC) NMR spectra were recorded at 27 °C on a Bruker Avance III 600 MHz NMR spectrometer (Bruker Corporation (Billerica, MA, USA)). ^1^H chemical shifts were referenced to residual HDO, with δ 4.78 ppm (27 °C) as the internal standard. ^13^C chemical shifts were determined in relation to DSS (δ 0.00 ppm) calibrated externally. COSY and HMQC were used to assign signals. HMBC and NOESY were used to assign inter-residue linkages and sequences [27].

### 2.9. In Vitro Experiments

RAW264.7 cells were used between passages 3–5 and cultured in RPMI-1640 medium containing 10% fetal bovine serum, 100 U/mL penicillin and 100 μg/mL streptomycin at 37 °C in 5% CO_2_. The cells were diluted to a final concentration of 2 × 10^5^ cells/mL in 24-well plates. FVPB1 samples were filtered using a 0.22-micron sterile membrane for sterilization. The cells were divided into three groups (*n* = 3): negative control (ND, treated with PBS), Ox-LDL (HFD, treated with 20 μg/mL Ox-LDL), and sample groups (Ox-LDL group treated with 100, 200, or 400 μg/mL FVPB1). In sample groups, 20 μg/mL Ox-LDL (Beijing Biosynthesis Biotechnology Co., Ltd., Beijing, China) and FVPB1 were co-cultured with cells for 24 h. According to the manufacturer’s protocol of the Oil Red O Staining Kit, the air-dried cells were washed twice with PBS, stained with Oil Red O solution for 15 min, then washed with distilled water for 20 s, and finally mounted with an aqueous mounting medium. Images were acquired using a fluorescence microscope (Axio Observer 3, ZEISS, Jena, TH, Germany).

### 2.10. Zebrafish Experiments

The AB strain zebrafish embryos obtained by natural mating were purchased from Nanjing YSY Biotechnology Co., Ltd. (Nanjing, China) and cultured in fish water (aerated for 48 h) for 7 days. The 7-day-old zebrafish were divided into groups (*n* = 10/group): natural diet (ND), HFD (treated with 0.1% egg yolk powder), and treatment groups (HFD treated with 100, 200, or 400 μ g/mL FVPB1) and placed into 12-well culture plates. The zebrafish were randomly divided into groups of 10 fish per well, with three wells in each group. Three independent replicate experiments were conducted, with light conditions of 12 h of light/12 h of darkness. In HFD group and FVPB1 groups, 0.1% egg yolk (*w*/*v*) with or without FVPB1 were co-cultured with zebrafish for 36 h at 28 °C. After the treatment, zebrafish larvae were fixed with 4% paraformaldehyde for tissue fixation followed by dehydration with methanol at 25%, 50%, 75%, and 100% concentrations diluted in PBS. Each group was stained with Oil Red O for 2 h, rehydrated with 100%, 75%, 50%, and 25% methanol diluted in PBS (15 min per wash), and sealed with a waterborne agent. The images were captured using a fluorescence microsco premagnification of 20× (Axio observer3) supplied by ZEISS of Germany. The total integrated optical density of lipids in zebrafish was measured using Image Pro Plus (IPP) software (version 6.0), and lipid accumulation was quantified as previously described [35].

### 2.11. Mouse Experiments

#### 2.11.1. Lipid-Lowering Model

Forty-eight specific pathogen-free male C57BL/6J mice (weighing 20 ± 2 g) were purchased from Jicui Yaokang Biotechnology Co. Ltd. (Jiangsu, China; permit SCXK (SU) 2018-0008). Shanghai Academy of Agricultural Sciences approved all animal protocols (No. SAASPZ0424110). This study was conducted per requirements for the protection of experimental animals. Mice were housed at Jiamu Biological Products Co. Ltd. (Shanghai, China, permit SYXK(HU) 2015-0007).

The high-fat diet consisted of 76.86% basal diet, 10% lard, 10% egg yolk powder, 1% cholesterol, 1.58% mineral, 0.45% vitamin, and 0.11% choline bitartrate. Both diets were obtained from Shanghai Chaorui Biotechnology Co., Ltd. (Shanghai, China). FVPB1 and simvastatin were administered by gavage in physiological saline at 10 mL/kg. Mice were randomly divided into six experimental groups (*n* = 8/group): ND, HFD, HFD plus FVPB1 (50 mg/kg/d FVPB1 [HFDL], 100 mg/kg/d FVPB1 [HFDM], and 200 mg/kg/d FVPB1 [HFDH]), and HFD plus 2.5 mg/kg/d simvastatin (SV). All mice were fed their respective diets for 6 weeks with ad libitum access to water. Mice were weighed every 3 days.

At the end of both week 3 and week 6, all mice underwent a 12-h fast prior to sample collection. Blood samples were then collected from the orbital sinus and centrifuged at 4000× *g* for 10 min. Serum was collected and stored at −80 °C. Feces were collected two days before the end of week 6 and stored at −80 °C for fecal index measurement. Following blood collection at the end of week 6, mice were euthanized by cervical dislocation. The livers were excised, rinsed, photographed, and weighed. Liver tissues were divided into three parts: one was mixed in PBS (0.1 g of liver to 9 mL of PBS) for liver index detection, another was fixed in 4% paraformaldehyde for liver histology analysis, and the third was snap-frozen in liquid nitrogen and stored at −80 °C for mRNA extraction. The intestinal segment was placed on ice, and the colon was excised. The caecum was opened, and the cecal contents were scraped and stored in a −80 °C freezer for sequencing. Body weight gain and hepatic index were calculated as follows:

Body weight gain (g) = body weight at the end of week 6 (g) − initial body weight (g).

Hepatic index (mg/g) = liver weight (mg)/body weight at the end of week 6 (g)

#### 2.11.2. Haematoxylin and Eosin (HE) Staining

Liver tissues were fixed in 4% paraformaldehyde solution for 24 h and embedded in paraffin for sectioning. Liver sections (5-μm thick) were stained with HE after deparaffinization. Images were acquired using a fluorescence microscope (Axio Observer 3, ZEISS, Jena, TH, Germany).

#### 2.11.3. Biochemical Analyses of Serum, Liver, and Feces Samples in Mice

The levels of TC (Cat No. A111-1-1), TG (Cat No. A110-1-1), LDL-C (Cat No. A113-1-1), HDL-C (Cat No.A112-1-1), serum apolipoprotein A-I (Cat No. E020-1-1), apolipoprotein B (Cat No. E021-1), liver HMGCR (Cat No. H236), liver human CYP7A1 (Cat No. H461-1-2), liver lecithin cholesterol acyltransferase (LCAT, Cat No. H237), and fecal coprostanol (Cat No. A111-1-1) and bile acids (Cat No. 003-2-1) were measured using assay kits (all Jiancheng Biological Engineering Institute, Nanjing, China) following the manufacturer’s instructions.

Liver tissues were divided into three parts: one for liver index detection was stored it in a −80 °C freezer, another was fixed in 4% paraformaldehyde for liver histology analysis, and the third was snap-frozen in liquid nitrogen and stored at −80 °C for mRNA extraction. Samples were aliquoted and frozen immediately after preparation, and each aliquot was thawed only once for use. Before liver index detection, 0.1 live tissue was mixed with 9 mL PBS including 1× protease inhibitor cocktail (Roche, South San Francisco, CA, USA, Cat No. 11836170001), and homogenized to prepare a liver tissue homogenate.

### 2.12. Reverse Transcription Quantitative Polymerase Chain Reaction (RT-qPCR)

Approximately 20 mg mouse liver sample were collected, and total RNA was extracted utilizing the RNA extraction kit (Qiushuang, Shanghai, China) following the manufacturer’s instructions. The extracted RNA was stored at −80 °C for future analysis. The concentration and purity of the RNA were assessed using 1 μL sample on a nucleic acid detector, and OD260/OD280 ratios between 1.8 and 2.1, indicating high purity. To assess RNA integrity, samples were run on a 1% agarose gel at 120 V for 30 min. Reverse transcription was performed according to the manufacturer’s instructions (Takara) as follows: genomic DNA removal at 42 °C for 2 min, followed by cDNA synthesis at 3 °C for 15 min, reaction termination at 85 °C for 5 s, and final storage of the product at 4 °C. Reverse transcription quantitative polymerase chain reaction (RT-qPCR) was performed using the SYBR Premix EX Taq™ qPCR kit (Takara Bio Inc., Kusatsu, Shiga, Japan) in accordance with the manufacturer’s protocol. The reaction mixture composition is detailed in Table 1. The thermal cycling protocol consisted of an initial denaturation step at 95 °C for 30 s, followed by 40 cycles of denaturation at 90 °C for 5 s, annealing at 60 °C for 10 s, and extension at 72 °C for 15 s. β-Actin was used as an internal reference gene for normalization of target gene mRNA expression. Relative gene expression levels were calculated using the 2^−ΔΔCt^ method based on cycle threshold (Ct) values obtained for both reference and target genes. The primer sequences are listed in Table 2 and were synthesized by Shenggong Bioengineering Co., Ltd. (Shanghai, China).

### 2.13. Gut Microbiota Analysis

The composition and structure of the gut microbiota were assessed using 16S rRNA sequencing by Personal Biotechnology Co. Ltd. (Shanghai, China). The V3-V4 regions of the 16S rRNA gene were amplified using primers F338 (5′-ACTCCTACGGGAGGCAGCA-3′) and R806 (5′-GGACTACHVGGGTWTCTAAT-3′). Following amplification, microbiome bioinformatics analysis was performed using QIIME2 version 2019.4. During this step, sequences underwent quality filtering and denoising before being assembled and screened for chimerism via the DADA2 plugin. This process resulted in a total of 1,798,905 valid sequences, with the highest sequence count per sample being 77,441 and the lowest 36,621. Notably, sequence lengths predominantly ranged between 400 and 450 bp. The structure and diversity of the gut microbiota were determined using the Illumina MiSeq platform following the manufacturer’s instructions. Venn diagrams were used to compare shared and unique amplicon sequence variants (ASVs) across mouse groups. Beta diversity was calculated based on the Bray–Curtis dissimilarity index, and differences in beta diversity between groups were evaluated using permutational multivariate analysis of variance (PERMANOVA).

### 2.14. Statistical Analysis

All experiments were carried out in triplicate and data were presented as either mean ± SD or mean ± SEM, depending on the type of analysis. For comparisons involving more than two groups, a one-way ANOVA followed by Tukey’s post hoc test was performed. Significant differences between groups are indicated in figures and tables using “*” or “^#^”. Statistical analyses and data visualization were performed using GraphPad Prism (version 8.0.2) and SPSS (version 27).

## 3. Results and Discussion

### 3.1. FVPB1 Structural Characteristics

**Figure 1:**
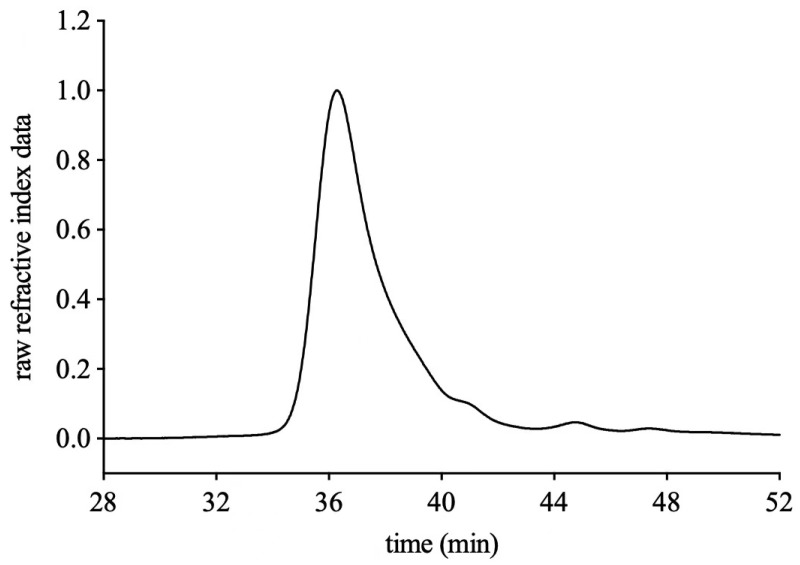
HPLC profile of FVPB1 using PWXL 6000 and 3000 gel filtration columns.

FVPB1 had a molecular weight of 2.04 × 10^4^ g/mol (Figure 1), total carbohydrate content of 95.26 ± 3.75%, and extraction yield of 0.07 ± 0.01% fruiting bodies. The Mw/Mn of FVPB1 is 2.17, indicating a linear backbone structure with minimal branching or uniformly distributed branch points. The BCA^TM^ protein assay confirmed that FVPB1 was protein-free. The monosaccharide molar ratio was Glu: Gal: Man: Fuc = 8.5: 2.2: 1.4: 1, reflecting a heterogeneous polysaccharide structure.

**Figure 2:**
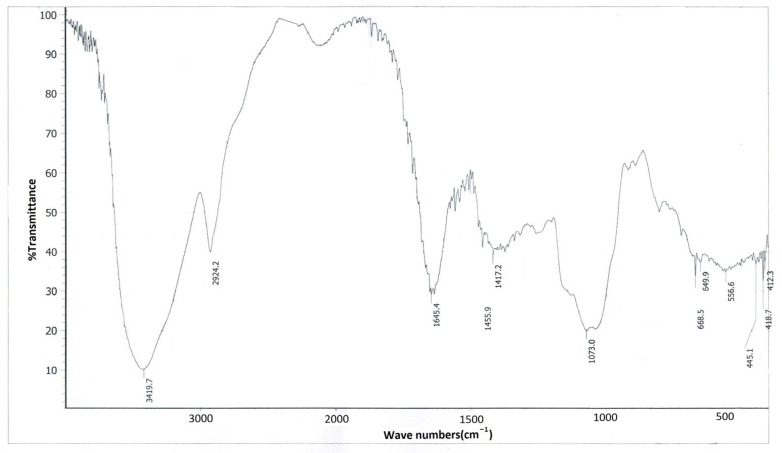
Infrared spectrogram of FVPB1.

In the infrared spectrum of FVPB1 (Figure 2), the 3419.7 cm^−1^ peak corresponded to the sugar ring’s hydroxyl stretching, 2924.2 cm^−1^ to C–H stretching, and 1645.4 cm^−1^ to water binding or C=O stretching. Three strong peaks at 1021, 1073 and 1182 cm^−1^ at 1000–1200 cm^−1^ (not labelled) were characteristic of pyranose glycosides, whereas peaks at 840 and 890 cm^−1^ (not labelled) suggested the presence of α- and β-pyranose glycosides in FVPB1 [36].

The methylation results in Table 1 show that the sugar subunits of FVPB1 were primarily linked as follows: terminal galactose and mannose; glucose in 1, 3 and 1, 4 forms; fucose in 1, 2 forms, glucose in 1, 3, 6 form (main chain); and additional terminal glucose residues (Table 3).

**Figure 3:**
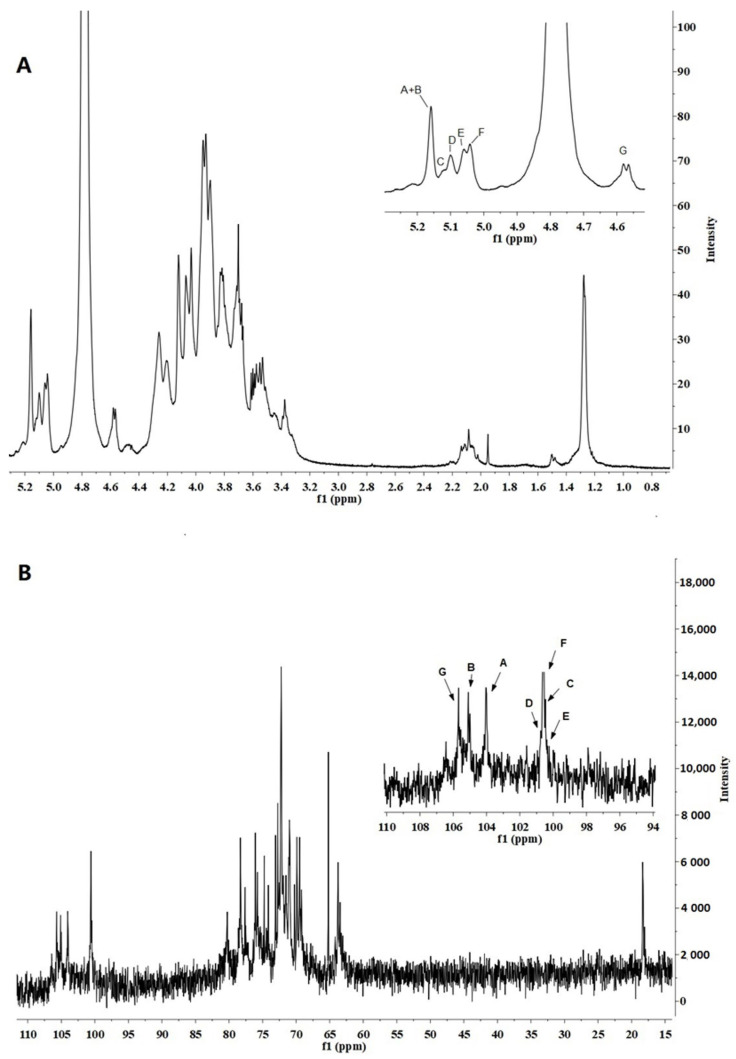
500 MHz ^1^H NMR and 125 MHz ^13^C NMR spectrum of FVPB1 polysaccharide in deuterium oxide (D_2_O) at 25 °C. The anomeric protons are labelled as (A–G). (**A**). 500 MHz ^1^H NMR spectrum of FVPB1polysaccharide in D_2_O at 25 °C. The anomeric protons are labelled as (A–G). (**B**) 125 MHz ^13^C NMR spectrum of FVPB1 polysaccharide in D_2_O at 25 °C.

The ^1^H NMR (500 MHz) spectra of FVPB1 (Figure 3A) showed seven anomeric proton signals between δ4.57 and δ5.17. The chemical shifts were δ5.17, δ5.16, δ5.12, δ5.11, δ5.10, δ5.06, and δ4.57 (*J*_H-1,H-2_ = 9 Hz), corresponding to residues A, B, C, D, E, F, and G from low to high field. The signal peak at δ1.28 was indicative of an oxymethyl, whereas the other hydrogen signals ranged between δ3.30 and δ4.50. The ^13^C NMR spectrum identified seven primary anomeric carbon signals in the end group carbon region, with chemical shifts of δ105.70, δ105.01, δ104.05, δ100.65, δ100.63, δ100.48, and δ100.38 (Figure 3B). The peak at δ1.28 in the ^1^H NMR spectrum corresponded to a carbon signal at δ18.39 according to the Heteronuclear Single Quantum Coherence spectrum, suggesting it may represent the sixth carbon of deoxyglucose. Monosaccharide composition analysis confirmed the presence of fucose.

A two-dimensional NMR analysis was used to further illustrate the structure of the FVB1 subunit. The anomeric proton signal at δ5.17 and small *J*_H-1,H-2_ values indicated that residue A was an α-linked residue. H-1, H-2, and H-3 of residue A were inferred from the ^1^H-^1^H Correlation Spectroscopy (COSY), whereas H-4 could only be inferred from the Total Correlation Spectroscopy (TOCSY). Although H-5 signals initially went undetected, the Nuclear Overhauser Effect Spectroscopy (NOESY) data revealed a cross-peak between H-4 and H-5, confirming H-5’s presence. Further analysis of the TOCSY spectrum revealed H-5/H-6a, H-5/H-6b, H-6a, and H-6b. Heteronuclear Multiple Bond Correlation (HMBC) spectra showed peaks for H-1, C-3, and C-5, placing H-5 and H-6 on residue A. In the TOCSY spectrum, the spin-coupled system could only be extrapolated from H-1 to the smaller coupling constants between H-3 and H-4/H-5, indicating that residue A was in a galactose configuration [37]. The notable shift in C-1 to a lower field and the absence of a δ70–δ80 carbon signal suggested that residue A was α-D-Gal*p*-(1→.

Analysis of the ^1^H-^1^H COSY and TOCSY spectra enabled the assignment of hydrogen chemical shifts for residues B, C, D, and G (Table 4). Clear cross-peaks in the COSY spectrum indicated a glucose configuration. Using HMQC data, corresponding carbon chemical shifts in the sugar ring were inferred. The ^1^H NMR revealed that the chemical shifts in H-1 residues B (δ5.16), C (δ5.12), and D (δ5.11) exceeded δ4.8, with *J*_H-1,H-2_ < 3 Hz, confirming these as α configurations, whereas residue G (δ4.57) was in the β-configuration. Additionally, the chemical shifts for C-4 (δ79.21) of residue B, C-3 (δ77.61) and C-6 (δ69.49) of residue C, C-3 (δ80.22) of residue D, and C-3 (δ78.20) and C-6 (δ69.96) of residue G towards lower fields identified B as →4)-α-D-Glc*p*, C as →3,6)-α-D-Glc*p*, D as →3)-α-D-Glc*p*, and G → 3,6)-β-D-Glc*p*.

Residue E exhibited an anomeric proton signal at δ5.10 with a very small ^3^*J*_H-1,H-2_ value, signifying the presence of an α-linked residue. The resonances of H-1 for H-2, H-3, and H-4 in this residue were determined using COSY, TOCSY, and NOESY spectral data, respectively. Assignments for H-5 and H-6 were based on their ^1^H-^1^H COSY spectrum. Resonance peaks corresponding to H-4 and H-6 in the HMBC spectrum supported the assignments of H-5 and H-6 within residue E, respectively. A methyl proton signal at α 1.28 was consistent with the presence of a fuc*p* residue. The observed downfield shift for C-2 (δ76.04) of sugar residue E further supported its identification as →2)-α-L-Fuc*p*.

For residue F, the anomeric proton resonates at δ5.06, with *J*_H-1,H-2_ < 3 and *J*_H-4,H-5_ = 8. Strong cross-peaks between H-1 and H-2 in the NOESY spectrum confirmed a D-Man*p* configuration. However, since *J*_H-1,H-2_ < 3 is not definitive for determining the α or β configuration in mannose, the chemical shifts in H-5 and C-5 were compared with reported values. According to Jansson et al. [38], H-5 (δ3.82) and C-5 (δ73.34) indicate an α configuration, whereas H-5 (δ3.38) and C-5 (δ77.00) correspond to a β configuration; thus, residue F was assigned as α-D-Mannose. Additionally, the notable shift in C-1 to a lower field and the absence of carbon signals between δ70 and δ80 suggest that residue F is α-D-Man*p*-(1→, consistent with Senchenkova et al. [39].

The connection modes and sequences between each sugar residue in the FVPB1 repeat unit were determined and verified using HMBC and NOESY spectra (Table 5). The NOESY spectrum showed correlations between H-1/residue A and H-6/residue C, H-1/residue B and H-3/residue C, H-1/residue B and H-3/residue D, H-1/residue C and H-4/residue B, H-1/residue C and H-2/residue E, H-1/residue D and H-3/residue C, H-1/residue E and H-6/residue G, H-1/residue F and H-3/residue G, and H-1/residue G and H-4/residue B.

The HMBC spectrum revealed the following correlations: H-1/sugar residue A and C-6/residue C, H-1/residue B and C-3/residue D, H-1/residue C and C-2/residue E, H-1/residue E and C-6/residue G, H-1/residue F and C-3/residue G, and H-1/residue G and C-4/residue B (Table 6).

**Figure 4:**
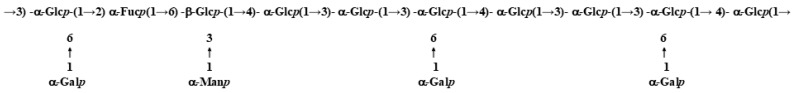
Repeat union of FVPB1.

From the data from Table 1, Table 2, Table 3 and Table 4, combined with NMR spectra, the sugar repeat units of FVPB1 were determined (Figure 4).

### 3.2. FVPB1 Inhibits Lipid Accumulation in RAW264.7 Cells and HFD-Induced Zebrafish Larvae

**Figure 5:**
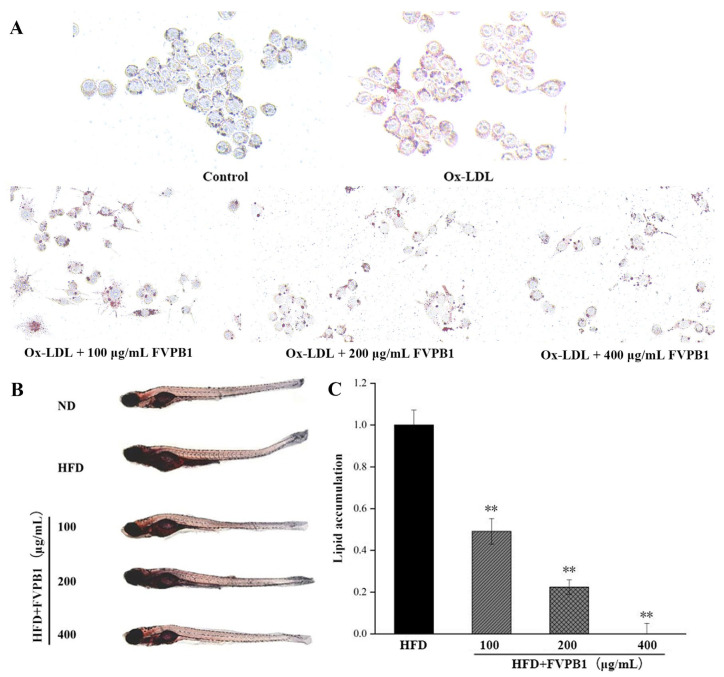
The effect of FVPB1 on lipid accumulation in RAW264.7 cells and zebrafish. (**A**) RAW264.7; (**B**) zebrafish; (**C**) Integrated optical density analysis of adipose accumulation in the blood vessels and stomach of zebrafish. Values are expressed as mean ± SD (*n* = 10). Statistical analysis was performed using one-way ANOVA followed by Tukey’s post hoc test. ** represents an extremely significant difference compared with HFD (*p* < 0.01). Four treatment groups were as follows: HFD, treated with 0.5% egg yolk powder group; HFD + 100 μg/mL FVPB1, HFD treated with 100 μg/mL FVPB1 group; HFD + 200 μg/mL FVPB1, HFD treated with 200 μg/mL FVPB1 group; HFD + 400 μg/mL FVPB1, HFD treated with 400 μg/mL FVPB1group.

RAW264.7 from the HFD group treated with 20 μg/mL ox-LDL exhibited abundant lipid droplets along the inner side of the cell membrane, exhibiting a ring-like distribution (Figure 5A). After adding 100, 200, or 400 μg/mL FVPB1 to co-cultures with RAW264.7, the number of lipid droplets decreased and became less tightly clustered against the cell membrane, exhibiting a more granular distribution. The strongest effect was elicited by 400 μg/mL FVPB1.

Treatment with FVPB1 reduced vascular and stomach fat accumulation in a dose-dependent manner (Figure 5B). Lipid accumulation was significantly decreased by 51%, 78%, and 100% at FVPB1 concentrations of 100, 200, and 400 μg/mL, respectively (*p* < 0.01; Figure 3C), demonstrating the significant lipid-lowering effect of FVPB1 in zebrafish larvae.

### 3.3. FVPB1 Lowers Lipid Levels in HFD-Mouse Models

#### 3.3.1. FVPB1 Inhibits Weight Gain and Repairs Liver Injury in HFD-Induced Mouse

**Figure 6:**
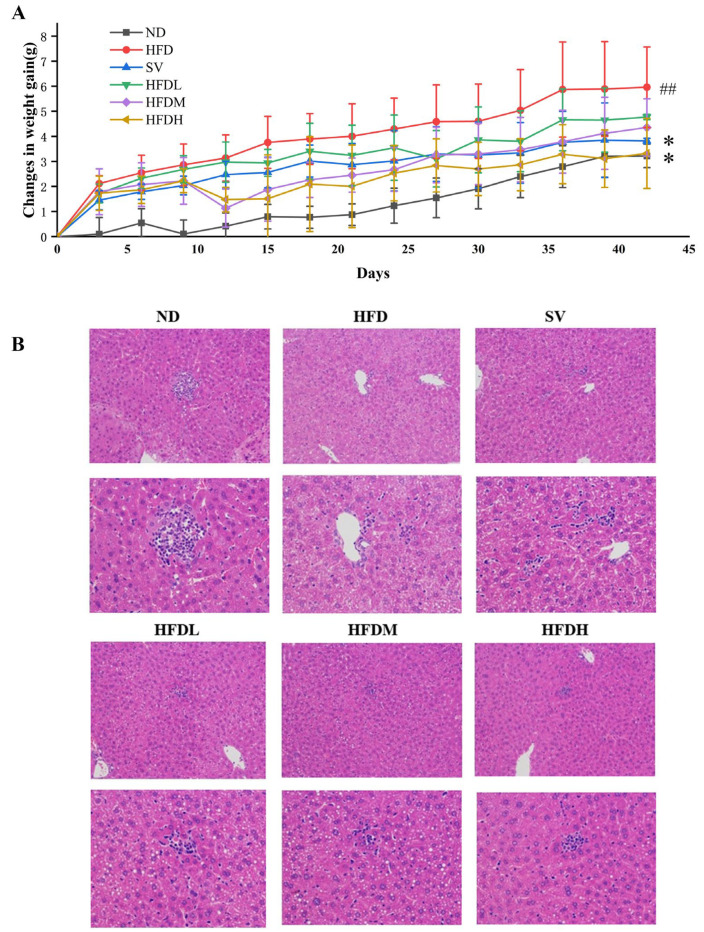
The effect of FVPB1 on weight gain and liver injury in HFD-induced mouse. (**A**) Weight gain of mice in each group over 42 days; (**B**) HE staining of liver (magnification: first column, 200×; second column, 400×). Values are expressed as mean ± SD (*n* = 10). Statistical analysis was performed using one-way ANOVA followed by Tukey’s post hoc test. ## indicates an extremely significant difference between ND and HFD (*p* < 0.01); * denotes a significant difference compared with HFD (*p* < 0.05). Six treatment groups were as follows: ND, treated with a natural diet group; HFD, treated with a high-fat diet group; SV, HFD treated with 2.5 mg/kg/d simvastatin; HDFL, HFD treated with 100 μg/mL FVPB1; HDFM, HFD treated with 200 μg/mL FVPB1; HDFH, HFD treated with 400 μg/mL FVPB1.

The body weight of mice in all groups increased gradually over time (Figure 6A). At week 3, weight gain in the medium-dose group was significantly reduced by 41.80% compared with the HFD group. FVPB1 intervention at various doses resulted in varying degrees of reduced hepatic steatosis, fewer intracellular vacuoles, more regularly organized tissues, and less liver damage (Figure 6B). The HFDH group exhibited the most significant effects.

#### 3.3.2. FVPB1 Repairs the Levels of Lipid Correlated Indexes in the Serum and Liver of Hyperlipidemic Mice

**Figure 7:**
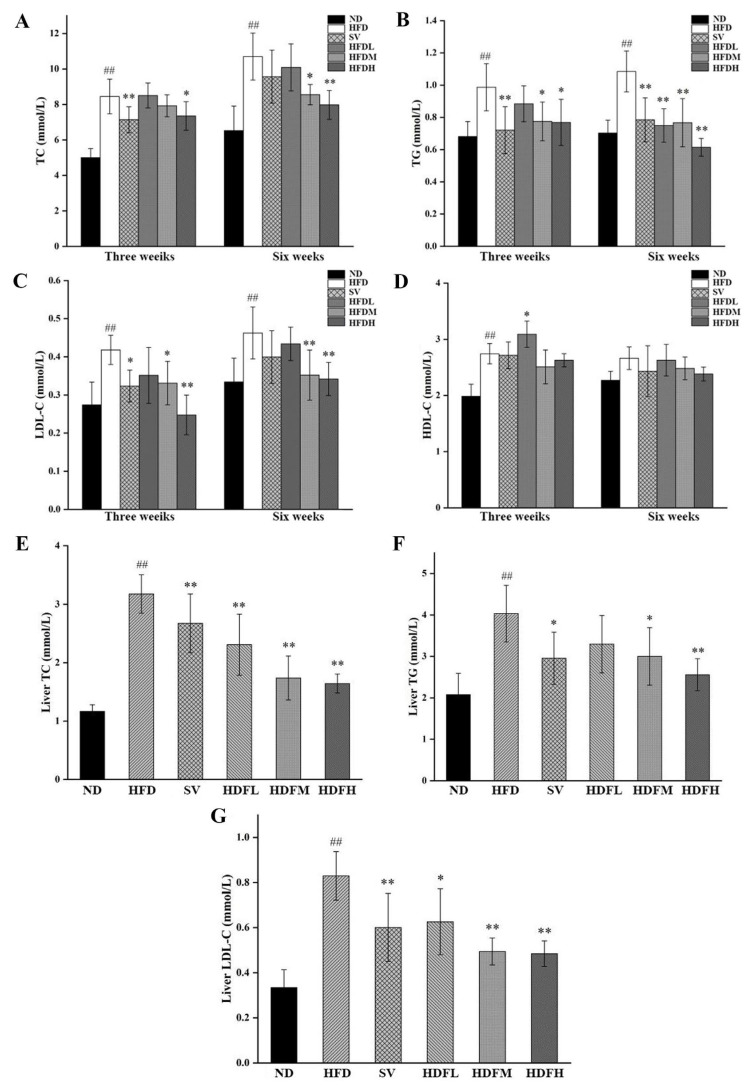
Serological and Liver lipid indicators of mice in each group. (**A**–**D**) Serum levels of TC, TG, LDL-C, and HDL-C in each group of mice; (**E**–**G**) Liver TC, TG, and LDL-C levels of mice in each group. Values are expressed as mean ± SD (*n* = 10). Statistical analysis was performed using one-way ANOVA followed by Tukey’s post hoc test. ## indicates an extremely significant difference between ND and HFD (*p* < 0.01); * denotes a significant difference compared with HFD (*p* < 0.05), while ** represents an extremely significant difference compared with HFD (*p* < 0.01). Six treatment groups were as follows: ND, treated with a natural diet group; HFD, treated with a high-fat diet group; SV, HFD treated with 2.5 mg/kg/d simvastatin; HDFL, HFD treated with 100 μg/mL FVPB1; HDFM, HFD treated with 200 μg/mL FVPB1; HDFH, HFD treated with 400 μg/mL FVPB1.

HFDM and HFDH groups exhibited TC reductions of 20.09% and 25.46% compared with the HFD group (*p* < 0.01), respectively, after 6 weeks (Figure 7A). At the end of weeks 3, the serum TG levels of mice in the HFDM and HFDH groups were significantly reduced by 21.49% and 22.13% (*p* < 0.05), respectively, compared with the HFD group (Figure 7B). After 6 weeks, the HFDL, HFDM, and HFDH groups showed significant reductions of 30.87%, 29.32%, and 43.37% (*p* < 0.01), further supporting the TG-lowering effect of FVPB1 (Figure 7B). After 3 weeks, the serum LDL-C levels in the SV, HFDM, and HFDH groups were significantly reduced by 22.61%, 15.92%, and 40.76%, respectively, compared with the HFD group (*p* < 0.01) (Figure 7C). After 6 weeks, the HFDM and HFDH groups exhibited 23.89% and 26.08% decreases, respectively, compared with the HFD group (*p* < 0.01) (Figure 7C). At the end of week 3, serum HDL-C levels in the HFDL group increased by 12.65% compared with the HFD group (*p* < 0.05) (Figure 7D). By the end of week 6, no significant differences were observed in the serum HDL-C levels across all groups (*p* > 0.05) (Figure 7D).

The hepatic TC levels in the HFDL, HFDM, and HFDH groups were reduced by 27.34%, 45.31%, and 48.31%, respectively, compared with the HFD group (*p* < 0.01) (Figure 7E). The hepatic TG levels in HFDM and HFDH groups decreased by 25.62% and 36.59%, respectively, compared with the HFD group (*p* < 0.05) (Figure 7F). Additionally, the hepatic LDL-C levels in the HFDL, HFDM, and HFDH groups decreased by 24.57% (*p* < 0.05), 40.41% (*p* < 0.01), and 41.64% (*p* < 0.01), respectively, compared with the HFD group (Figure 7G).

#### 3.3.3. FVPB1 Restores Abnormal Serum Apolipoprotein Levels Induced by HFD in Mice

**Figure 8:**
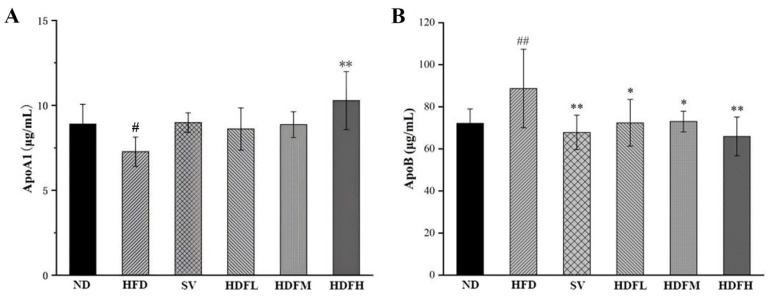
Effect of FVPB1 on the level of serum apolipoprotein in mice. (**A**) Apolipoprotein ApoA1. (**B**) Apolipoprotein ApoB. Values are expressed as mean ± SD (*n* = 10). Statistical analysis was performed using one-way ANOVA followed by Tukey’s post hoc test. # indicates a significant difference between ND and HFD (*p* < 0.05), while ## indicates an extremely significant difference between ND and HFD (*p* < 0.01); * denotes a significant difference compared with HFD (*p* < 0.05), while ** represents an extremely significant difference compared with HFD (*p* < 0.01). Six treatment groups were as follows: ND, treated with a natural diet group; HFD, treated with a high-fat diet group; SV, HFD treated with 2.5 mg/kg/d simvastatin; HDFL, HFD treated with 100 μg/mL FVPB1; HDFM, HFD treated with 200 μg/mL FVPB1; HDFH, HFD treated with 400 μg/mL FVPB1.

The HFDH group exhibited a significant 41.39% increase in serum ApoA1 levels compared with the HFD group (*p* < 0.01), indicating that FVPB1 effectively increased serum ApoA1 levels in HFD-fed mice (Figure 8A). All FVPB1 dose groups demonstrated substantial decreases in serum ApoB levels 18.41% (*p* < 0.05), 17.68% (*p* < 0.05), and 25.68% (*p* < 0.01), respectively, compared with the HFD group (Figure 8B).

#### 3.3.4. FVPB1 Restores the Expression Disorder of Cholesterol Transport-Related Factors in Mice Induced by HFD at the mRNA and Enzymes Level

**Figure 9:**
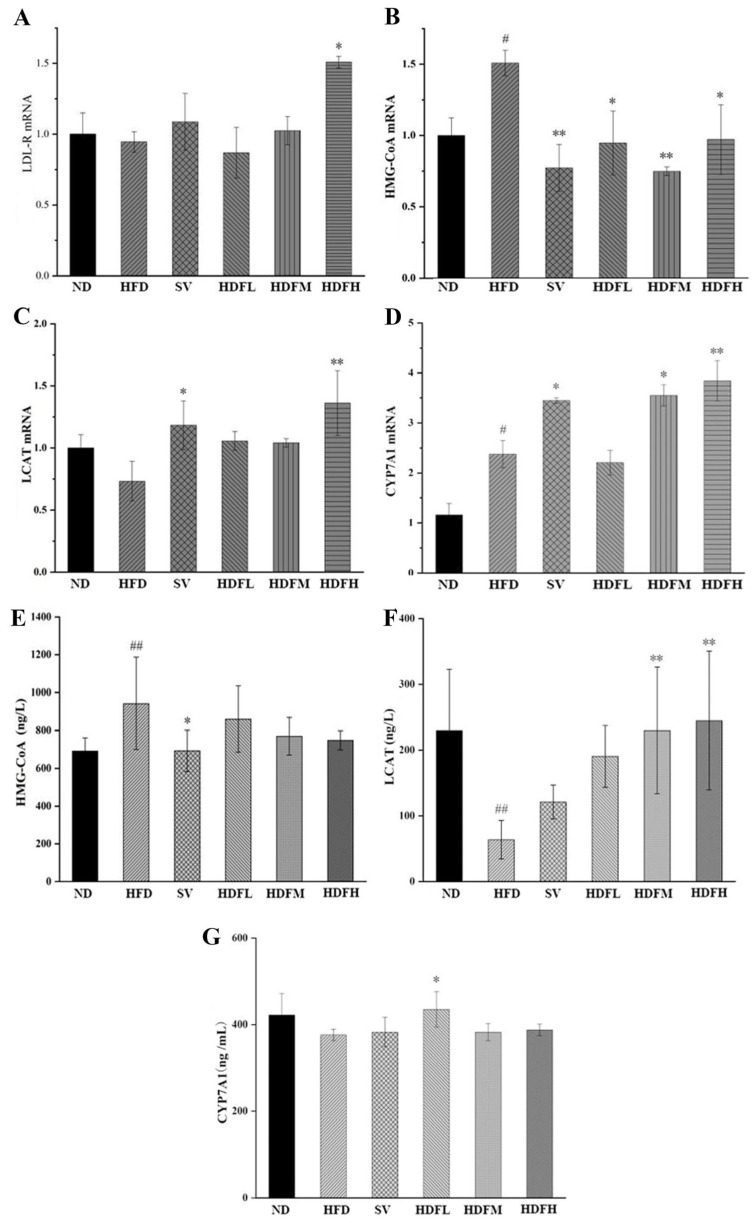
Effect of FVPB1 on the expression of cholesterol transport-related factors in mice induced by HFD at the mRNA and enzymes level. (**A**–**D**) Ldlr mRNA, Hmgcr mRNA, Lcat mRNA, and Cyp7a1 mRNA; (**E**–**G**) HMG-CoA reductase, LCAT enzyme, and CYP7A1 enzyme. Values are expressed as mean ± SD (*n* = 10). Statistical analysis was performed using one-way ANOVA followed by Tukey’s post hoc test. # indicates a significant difference between ND and HFD (*p* < 0.05), while ## indicates an extremely significant difference between ND and HFD (*p* < 0.01); * denotes a significant difference compared with HFD (*p* < 0.05), while ** represents an extremely significant difference compared with HFD (*p* < 0.01). Six treatment groups were as follows: ND, treated with a natural diet group; HFD, treated with a high-fat diet group; SV, HFD treated with 2.5 mg/kg/d simvastatin; HDFL, HFD treated with 100 μg/mL FVPB1; HDFM, HFD treated with 200 μg/mL FVPB1; HDFH, HFD treated with 400 μg/mL FVPB1.

*Ldlr* expression in the HFDH group was significantly increased by 59.57% compared with the HFD group (*p* < 0.05), suggesting that FVPB1 can upregulate *Ldlr* expression, promote LDL-C transport, and reduce serum LDL-C levels (Figure 9A). The decreases of 37.22% (*p* < 0.05), 50.38% (*p* < 0.01), and 35.6% (*p* < 0.05) were observed in the HFDL, HFDM, and HFDH groups, respectively (Figure 9B). Compared with the HFD group, the hepatic *Lcat* mRNA expression in the SV group was significantly increased by 61.59% (*p* < 0.05), whereas high-dose FVPB1 significantly upregulated *Lcat* mRNA expression by 85.79% (*p* < 0.01) (Figure 9C). Therefore, FVPB1 supplementation upregulated *Lcat* mRNA expression, reduced blood cholesterol levels, and improved hyperlipidemia in mice. The *Cyp7a1* mRNA expression was upregulated by 49.47% (*p* < 0.05) in the HFDM group and 61.83% (*p* < 0.01) in the HFDH group compared with the HFD mice (Figure 9D). These results suggest that FVPB1 promotes cholesterol metabolism by enhancing *Cyp7a1* mRNA expression in mice.

The HFDL, HFDM, and HFDH groups also showed reduced hepatic HMGCR levels in a dose-dependent manner; however, these reductions were not significantly different from those in the HFD group, likely due to shorter feeding duration (*p* > 0.05) (Figure 9E). FVPB1 supplementation significantly increased LCAT levels in a dose-dependent manner. The HFDM and HFDH groups showed 2.6- and 2.8-fold increases compared with the HFD group (*p* < 0.01) (Figure 9F), indicating improved cholesterol reverse transport. In the HFDL group, CYP7A1 expression was increased by 15.6% compared with the HFD group (*p* < 0.05) (Figure 9G). However, significant differences were not observed between the HFDM and HFDH groups compared with the HFD group, likely due to negative feedback regulation of bile acids. That is, excessive bile acid content in the liver may reduce bile acid synthesis.

#### 3.3.5. FVPB1 Promotes the Production of Cholesterol Metabolites in Mice

**Figure 10:**
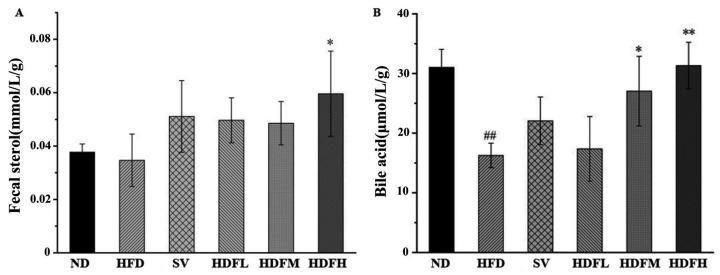
Effect of FVPB1 on cholesterol metabolites. Mouse fecal sterol (**A**) and bile acid (**B**). Values are expressed as mean ± SD (*n* = 10). Statistical analysis was performed using one-way ANOVA followed by Tukey’s post hoc test. ## indicates an extremely significant difference between ND and HFD (*p* < 0.01); * denotes a significant difference compared with HFD (*p* < 0.05), while ** represents an extremely significant difference compared with HFD (*p* < 0.01). Six treatment groups were as follows: ND, treated with a natural diet group; HFD, treated with a high-fat diet group; SV, HFD treated with 2.5 mg/kg/d simvastatin; HDFL, HFD treated with 100 μg/mL FVPB1; HDFM, HFD treated with 200 μg/mL FVPB1; HDFH, HFD treated with 400 μg/mL FVPB1.

The cecal steroid levels were elevated in groups receiving different FVPB1 doses compared with the HFD group, with the HFDH group exhibiting a significant 71.98% increase (*p* < 0.05) (Figure 10A). FVPB1 administration increased fecal sterol levels, suggesting that it promotes cholesterol excretion. This aligns with the observed reduction in hepatic cholesterol content. The HFDM and HFDH groups exhibited significant increases of 66.2% and 92.57%, respectively, compared with the HFD group (*p* < 0.05 and 0.01, respectively) (Figure 10B). Therefore, FVPB1 enhances bile acid excretion in feces, helping maintain cholesterol homeostasis.

#### 3.3.6. FVPB1 Restores the Structural Disorder of the Gut Microbiota in Mice Induced by HFD

##### Analysis of Venn Diagram and Beta Diversity

**Figure 11:**
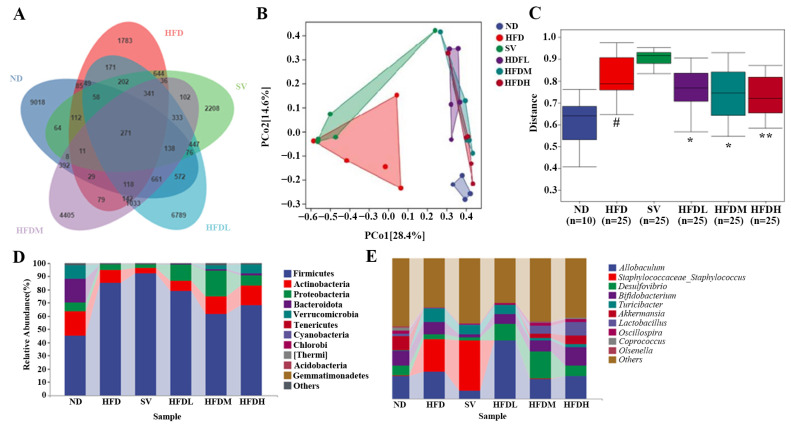
Effect of FVPB1 on the structural disorder of the gut microbiota in mice induced by HFD. (**A**) Venn diagram; (**B**) PCoA; (**C**) Statistical analysis of the PCoA by PERMANOVA; Bar plot showing the relative abundance of gut microbiota at phylum (**D**) and genus (**E**) levels. # indicates an extremely significant difference between ND and HFD (*p* < 0.01); * denotes a significant difference compared with HFD (*p* < 0.05), while ** represents an extremely significant difference compared with HFD (*p* < 0.01). Six treatment groups were as follows: ND, treated with a natural diet group; HFD, treated with a high-fat diet group; SV, HFD treated with 2.5 mg/kg/d simvastatin; HDFL, HFD treated with 100 μg/mL FVPB1; HDFM, HFD treated with 200 μg/mL FVPB1; HDFH, HFD treated with 400 μg/mL FVPB1.

The ND and HFD groups had 684 ASVs, whereas the SV and ND groups had 738 (Figure 11A). After FVPB1 intervention, the number of shared ASVs among the HFDL, HFDM, HFDH, and ND groups was 1077, 1575, and 1628, respectively, showing that HFD decreased gut microbiota similarity compared with ND. In PCoA analysis, PCo1 and PCo2 accounted for 28.4% and 14.6% of variance, respectively. The HFD and ND groups showed clear separation, whereas shorter distances were observed between the FVPB1-intervention and ND groups (Figure 11B). Additionally, HFD samples were more dispersed, reflecting considerable individual differences. As shown in Figure 11C, intergroup comparisons of Beta diversity, assessed using the Bray–Curtis distance metric, revealed significant differences between the ND and HFD groups (*p* < 0.05). Significant differences were also observed between the HFD and HFDL groups, as well as between the HFD and HFDM groups (*p* < 0.05). Moreover, a highly significant difference was detected between the HFD and HFDH groups (*p* < 0.01).

##### Structural Analysis at the Level of Phylum and Genus

Ten main phyla were identified: Firmicutes, Actinobacteria, Proteobacteria, Bacteroidota, Verrucomicrobia, Tenericutes, Cyanobacteria, Chlorobi, Thermi, Acidobacteria, and Gemmatimonadetes (Figure 11C). Notable differences were observed among the sample groups. For example, the relative abundance of Bacteroidota was high in the ND group and nearly undetectable in the HFD group. Following FVPB1 intervention, the proportion of Bacteroidota increased significantly. The top ten genera with the highest relative abundances included *Allobaculum*, *Staphylococcaceae_Staphylococcus*, *Desulfovibrio*, *Turicibacter*, *Akkermansia*, *Lactobacillus*, and *Oscillospira* (Figure 11D). The relative proportions of *Bifidobacterium* and *Lactobacillus* in the HFD group were lower than in the ND group, whereas *Staphylococcaceae_Staphylococcus* was higher. In contrast, the relative abundances of *Bifidobacterium* and *Lactobacillus* increased, while *Staphylococcaceae_Staphylococcus* decreased in the HFDH group. The Firmicutes to Bacteroidetes (F/B) ratio was included in this analysis. Due to substantial inter-individual heterogeneity in gut microbiota composition, the data exhibited wide variation across groups. However, the mean values indicate that FVPB1 intervention modulated the gut microbiota F/B ratio in a manner consistent with the trend observed in the ND group (Table 7).

Excessive fat intake can lead to hepatic fat deposition and related diseases, such as hepatitis, steatosis, fatty liver cirrhosis, and liver cancer [40]. Reducing lipid droplet accumulation in the liver helps alleviate lipid metabolic disorders. In the current study, FVPB1 administration lowered the liver index and hepatic lipid levels to normal levels in HFD-fed mice, consistent with previous reports [22].

LDL-C, the primary cholesterol carrier in serum, is associated with increased cardiovascular disease risk [41], HDL-C, an anti-atherosclerotic lipoprotein, helps transport cholesterol from extrahepatic tissues to the liver [42]. We hypothesize that FVP supplementation affects TG and TC transport by modulating serum and liver LDL-C levels. ApoB, found in LDL and VLDL, mediates cholesterol delivery to peripheral tissues [43]. FVPB1 lowers LDL-C levels by modulating ApoB levels. Therefore, FVP supplementation may offer a promising approach for cardiovascular disease management. HDL is a complex composed of lipids and proteins [44]. HDL-C refers to the amount of cholesteryl esters carried in the core of HDL particles, representing a static “inventory” concept [45]. In contrast, ApoA1 is the primary structural protein forming the shell of HDL particles and directly reflects the number of HDL particles in circulation [46]. This study found that at week 6, the high HFDH group showed no significant change in HDL-C levels, but ApoA1 levels significantly increased. This indicates that the high-concentration FVPB1 intervention led to an increase in the number of HDL particles, while the amount of HDL-C transported per HDL particle decreased, which is one of the key reasons why the total HDL-C level remained unchanged. LCAT, an enzyme synthesized primarily by the liver that adsorbs onto HDL particles, functions to esterify free cholesterol into cholesteryl esters and package them into the core of HDL particles [47]. This study found that at week 6, medium- and high-concentration FVPB1 interventions increased the quantity of LCAT enzyme but did not enhance its activity, which constitutes another important reason for the lack of change in HDL-C. This study suggests that the lipid-lowering effect of FVPB1 may not be achieved through enhancing reverse cholesterol transport.

LDLR, LCAT, HMG-CoA, and CYP7A1 are involved in reverse cholesterol transport, which promotes the decomposition of cholesterol [48]. Chang et al. reported that tobacco polysaccharides inhibit lipid over-accumulation in HepG-2 cells by increasing *CYP7A1* mRNA expression [49]. *Sparassis latifolia* polysaccharides regulate the hepatic sterol metabolism pathway by reducing mRNA expression of HMGCR [50]. The research found that in the medium- and high-FVBP1 groups, mRNA expression of CYP7A1 was elevated, but protein expression of CYP7A1 only increased in the low-FVBP1 group. The discrepancy between mRNA and protein expression levels originates from the mRNA translation process, as the translation process is influenced by post-transcriptional regulation, translational regulation, and post-translational regulation [51]. Additionally, we observed increases in fecal sterol and bile acid in the high-concentration FVBP1 group and the medium- to high-concentration FVBP1 groups, respectively, suggesting that the elevated cholesterol levels in this research may be related to enhanced CYP7A1 enzyme activity rather than CYP7A1 protein expression, with the changes in enzyme activity aligning with the mRNA expression levels. Furthermore, we found that the lipid-lowering effect of FVBP1 was accompanied by an increase in bile acid levels, suggesting that the lipid-lowering mechanism of bile acids may be achieved through activating the TGR5 receptor to promote thermogenesis and energy expenditure [52], modulating the FXR receptor to improve hepatic lipid metabolism [53], and altering the gut microbiota composition to indirectly regulate metabolism [54].

Hu et al. found that feeding *Ganoderma lucidum* polysaccharide GL55 to mice increased the relative abundance of Bacteroidota, especially *Prevotella*, in the gut and was inversely correlated with TC, TG, and free fatty acid levels in mice [55]. Xu et al. [56] reported that *Lentinula edodes* polysaccharide L2 increased the relative abundances of Proteobacteria and Actinobacteria in the small intestine, caecum, colon, and other parts of mice compared with the ND group. Similarly, the current study found that FVPB1 markedly increased the proportion of Proteobacteria and Actinobacteria in the gut microbiota of mice.

The phylum Proteobacteria is not exclusively a “repository for harmful bacteria”; it encompasses an immense functional diversity. An increase in its abundance at the phylum level should not be simplistically interpreted as an indicator of “impaired gut health.” As one of the largest bacterial phyla, Proteobacteria includes not only well-known pathogens (such as pathogenic strains of *Escherichia coli*, *Salmonella*, and *Helicobacter pylori*) but also numerous commensal species (e.g., non-pathogenic *E. coli* commonly found in the gut) and even some conditionally beneficial taxa (such as certain *Acetobacter* species and some relatives of bifidobacteria). In this study, we observed that the abundance of Proteobacteria followed a pattern of initial increase followed by a decrease across low-, medium-, and high-dose FVPB1 treatment groups. A similar trend was noted for *Desulfovibrio*, a genus within Proteobacteria. Notably, the high-dose FVPB1 group showed significantly lower abundances of both Proteobacteria and *Desulfovibrio* compared to the low-dose group. Since high-dose FVPB1 demonstrated superior lipid-lowering efficacy compared to the medium dose in the mouse model, these findings suggest that low and medium concentrations of FVPB1 may induce a stress-responsive increase in Proteobacteria and *Desulfovibrio*. In contrast, when the concentration of FVPB1 is elevated beyond a certain threshold, their abundance declines, correlating with enhanced lipid reduction. This indicates a negative association between the levels of Proteobacteria/*Desulfovibrio* and the lipid-lowering effect of FVPB1 observed in this study.

*Akkermansia* is a key bacteria linked to hyperlipidemia that can regulate adipocyte metabolism [57] and convert indigestible polysaccharides into short-chain fatty acids like acetate and butyrate, helping regulate lipid homeostasis [58]. In the current study, FVPB1 significantly increased the relative abundance of *Akkermansia* in the gut microbiota compared to the HFD group. *Lactobacillus*, another major probiotic, supports lipid metabolism and energy consumption. Chen et al. [59] reported that dietary intervention with *Lactobacillus* significantly reduced body weight and fat accumulation in obese mice after three weeks, improved liver steatosis, and regulated the gut microbiota, increasing the abundance of beneficial bacteria, such as *Bifidobacterium* and *Lactobacillus*, and reducing harmful bacteria, such as *Ruminococcus*.

*Bifidobacterium*, a common probiotic in the gut, has been widely researched regarding its potential lipid-lowering properties. An et al. [60] reported that an HFD led to a notable decrease in the number of *Bifidobacterium* species in obese rats, whereas *Lactobacillus* diet intervention improved the blood lipid levels, potentially through intestinal colonization, appetite regulation, and alleviation of liver steatosis. Additionally, *Bifidobacteria* improved intestinal epithelial cell barrier function and intestinal permeability in individuals with obesity, reduced intestinal endotoxin levels, and enhanced metabolism related to inflammatory reactions [61].

Grossi et al. [62] observed an increased prevalence of *Staphylococcus* spp. in overweight children and women compared to the general population, consistent with findings of elevated *Staphylococcus* in the HFD group of the current study. High-molecular-weight polysaccharides are not readily digested or absorbed by the body but can be broken down by certain beneficial intestinal bacteria to produce short-chain fatty acids, repair intestinal epithelial cell barriers, and improve lipid metabolism disorders. Additionally, short-chain fatty acids maintain an acidic environment in the intestine, supporting the growth of beneficial bacteria.

## 4. Conclusions

In this study, FVPB1 reduced lipid accumulation in Raw264.7 cells in vitro and zebrafish. Meanwhile, FVPB1 repaired liver injury and lipid metabolism disorders in HFD-fed mice, potentially by upregulating LCAT, LDL-R, and CYP7A1, and downregulating HMG CoA. Overall, FVPB1 could improve hyperlipidemia in model animals, likely through a lipid-lowering mechanism closely associated with the changes in the gut microbiota. This supports the use of *F. velutipes* polysaccharides in hypolipidemic functional food or healthcare products.

A brief limitations statement: This study has several limitations that should be acknowledged. First, the inconsistency in sampling timepoints between experimental groups may introduce confounding variation in the microbiome data. Second, although we observed significant shifts in microbial composition, we did not measure functional metabolites such as short-chain fatty acids (SCFAs) or bile acids, which limits our mechanistic interpretation of the host phenotypes. Third, while we identified several microbial taxa that changed in abundance, formal statistical correlations between these taxa and specific phenotypic outcomes were not explored and remain an important topic for future investigation. Finally, the sequencing data generated in this study have not yet been deposited in a public repository due to SAAS review board approval pending but will be made available prior to formal publication.

## Figures and Tables

**Table 1:** RT-qPCR reaction system.

Component	Volume (μL)
SYBR Premix Ex Taq II (Tli RNaseH Plus)	10
F Primer	0.4
R Primer	0.4
ROX Reference Dye (50X)	0.4
ddH_2_O	6.8
cDNA	2

**Table 2:** Primer design.

Gene	Primer Sequence (Forward)	Primer Sequence (Reverse)
ACTB	GTGCTATGTTGCTCTAGACTTCG	ATGCCACAGGATTCCATACC
HMG-CoA	GGAAACTCATGAACGTGGTGTG	TCCGATCACATTCTCACAGCAA
LCAT	GTTCCTCCGTTCAAACATTGGG	TTTCTCTGGGAGTGACATGACG
CYP7A1	GGCATCTCAAGCAAACACCATT	CTGGCAGGTTGTTTAGTTGCTC
LDL-R	TCAGTCCCAGGCAGCGTATC	CTTGATCTTGGCGGGTGTTC

**Table 3:** Linkage analysis of FVPB1 using GC-MS.

Methylated Sugar	Linkage	Fractions (%)
2, 3, 4, 6-Me_4_-Glc*p*	T-Glc*p*	4
2, 3, 4, 6-Me_4_-Gal*p*	T-Gal*p*	19
2, 3, 6-Me_3_-Glc*p*	1,4-Glc*p*	15
2, 4-Me_2_-Glc*p*	1,3,6-Glc*p*	29
2, 4, 6-Me_3_-Glc*p*	1,3-Glc*p*	21
3, 4, 6-Me_3_-Fuc*p*	1,2-Fuc*p*	7
2, 3, 4, 6-Me_4_-Man*p*	T-Man*p*	5

Me_4_(Tetra-O-methyl). Me_3_(Tri-O-methyl). Me_2_(Di-O-methyl). T (terminal).

**Table 4:** ^1^H and ^13^C NMR chemical shifts (ppm) of FVPB1 at 27 °C.

Residue	Proton or Carbon					
	H-1/C-1	H-2/C-2	H-3/C-3	H-4/C-4	H-5/C-5	H-6/C-6
Aα-Gal*p* (1→	5.17	3.97	4.04	3.82	4.13	3.83 ^a^, 3.93 ^b^
	104.05	69.30	74.34	76.12	70.96	63.71
B→4)-α-Glc*p* (1→	5.16	4.14	3.94	3.83	3.74	3.93 ^a^, 4.02 ^b^
	105.01	72.65	72.17	79.21	69.38	63.78
C→3,6)-α-Glc*p*-(1→	5.12	3.84	3.70	3.95	4.26	3.75 ^a^, 3.84 ^b^
	100.48	76.12	77.61	73.12	71.53	69.49
D 3→α-Glc*p*-(1→	5.11	3.86	4.05	3.80	4.08	3.68 ^a^, 3.75 ^b^
	100.65	72.23	80.22	70.09	72.34	65.10
E 2→α-Fuc*p* (1→	5.10	3.88	4.03	4.13	4.22	1.28
	100.38	76.04	71.92	72.66	69.87	18.39
F α-Man*p*-(1→	5.06	3.90	4.27	3.74	4.08	3.80 ^a^, 3.94 ^b^
	100.63	76.04	71.50	69.43	72.40	63.72
G→3,6)-β-Glc*p*-(1→	4.57	3.38	3.54	3.95	3.73	4.23 ^a^, 4.31 ^b^
	105.70	75.77	78.20	72.24	69.26	69.96

^a^ Chemical shift for H-6a. ^b^ Chemical shift for H-6b.

**Table 5:** Inter-glycosidic correlations of FVPB1 based on NOESY spectra.

Residue	Proton	Proton Correlation
A α-Gal*p* (1→	5.17(H-1)	**3.75(C:H-6)**, **3.84(C:H-6)**, 3.93(A:H-6), 3.97: (A:H-2)
B →4)-α-Glc*p* (1→	5.16(H-1)	**3.70(C:H-3)**, **4.05:(D:H-3)**, 3.94(B:H-3), 4.14(B:H-2)
	3.83(H-4)	**4.57(G:H-1)**
C →3,6)-α-Glc*p* (1→	5.12(H-1)	3.75(C:H-6), **3.83:(B:H-4), 3.88(E:H-2)**, 3.95(C:H-4)
	3.70(H-3)	**5.16(B:H-1), 5.11(D:H-1)**
	3.75 (H-6)	**5.17(A:H-1)**
D3→α-Glc*p* (1→	5.11(H-1)	**3.70(C:H-3)**, 4.05:(D:H-3)
	4.05(H-3)	**5.16(B:H-1)**
E 2→α-Fuc*p* (1→	5.10(H-1)	1.28(E:H-6), 3.88(E:H-2), 4.13(E:H-4), **4.23(G:H-6)**, **4.31(G:H-6)**
F α-Man*p* (1→	5.06(H-1)	**3.54(G:H-3)**, 3.74(F:H-4), 3.90(F:H-2), 4.08(F:H-5), 4.27(F:H-3)
G →3,6)-β-Glc*p* (1 →	4.57(H-1)	3.38(G:H-2), 3.54(G:H-3), **3.83(B:H-4)**
	3.54(H-3)	**5.06(F:H-1)**
	4.23,4.31(H-6)	**5.10(E:H-1)**

Inter-residue NOESY is shown in bold font.

**Table 6:** Inter-glycosidic correlations of FVPB1based on HMBC spectra.

Residue	Proton	Proton Correlation
A α-Gal*p* (1→	5.17(H-1)	76.12(A:C-4), **69.49(C:C-6)**
B →4)-α-Glc*p* (1→	5.16(H-1)	72.65(B:C-2), **80.22(D:C-3)**
C →3,6)-α-Glc*p*-(1→	5.12(H-1)	71.53(C:C-5), **76.04(E:C-2)**, 79.21(B:C-4)
	3.75, 3.84(H-6)	**104.05(A:C-1)**
D 3→α-Glc*p*-(1→	5.11(H-1)	65.10(D:C-6), 72.34(D:C-5)
	4.05(H-3)	**105.01(B:C-1)**
E 2→α-Fuc*p* (1→	5.10(H-1)	**69.96(G:C-6)**, 71.92(E:C-3)
	3.88(H-2)	100.48(C:C-1)
F α-Man*p*-(1→	5.06(H-1)	71.50(F:C-3), 72.40(F:C-5), **78.20(G:C-3)**
G →3,6)-β-Glc*p*	4.57(H-1)	75.77(G:C-2), 78.20(G:C-3), **79.21(B: C-4)**
	4.23, 4.31(H-6)	100.38(E:C-1)

Proton correlation HMBC spectra are shown in bold font.

**Table 7:** The Firmicutes/Bacteroidetes ratio of each group in mice.

Group	NFD	HFD	SV	HFDL	HFDM	HFDH
Firmicutes (%)	45.22 ± 14.14	84.95 ± 6.07	92.11 ± 1.44	79.13 ± 7.31	61.77 ± 16.55	68.40 ± 10.28
Bacteroidetes (%)	17.54 ± 11.55	0.04 ± 0.05	0.02 ± 0.02	0.26 ± 0.17	0.97 ± 0.0.75	1.94 ± 2.24
Firmicutes/Bacteroidetes ratio	4.07 ± 3.06	12,368.93 ± 13,311.48	10,212.24 ± 13,283.52	456.42 ± 283.05	114.8 ± 117.26	160.12 ± 199.61

## Data Availability

The original contributions presented in this study are included in the article. Further inquiries can be directed to the corresponding author.

## References

[1] Jameson J.L., Fauci A.S., Kasper D.L., Hauser S.L., Longo D.L., Loscalzo J. (2022). Harrison’s Principles of Internal Medicine.

[2] Goldstein J.L., Brown M.S. (2015). A century of cholesterol and coronaries: From plaques to genes to statins. Cell.

[3] World Health Organization (2022). Global Guidelines on Diet, Physical Activity, and Cardiovascular Disease Prevention.

[4] Rathore H., Prasad S., Sharma S. (2019). Medicinal importance of mushroom mycelia: Mechanisms and applications. J. Funct. Foods.

[5] Wasser S.P. (2014). Medicinal mushroom science: Current perspectives, advances, evidences, and challenges. Biomed. J..

[6] Ulrike L., Niedermeyer T.H.J., Jülich W.D. (2010). The pharmacological potential of mushrooms. Evid. Based Complement. Alternat. Med..

[7] Sokovi M., Glamolija J., Iri A., Petrovi J., Stojkovi D. (2018). Mushrooms as Sources of Therapeutic Foods. Therapeutic Foods.

[8] Wieczorek P.P., Witkowska D., Jasicka-Misiak I., Poliwoda A., Oterman M., Zielińska K. (2015). Bioactive alkaloids of hallucinogenic mushrooms. Stud. Nat. Prod. Chem..

[9] Sun Y.J., He H.Q., Wang Q., Yang X., Jiang S., Wang D. (2022). A Review of Development and Utilization for Edible Fungal Polysaccharides: Extraction, Chemical Characteristics, and Bioactivities. Polymers.

[10] Valverde M.E., Hernández-Pérez T., Paredes-López O. (2015). Edible mushrooms: Improving human health and promoting quality life. Int. J. Microbiol..

[11] Song X.L., Liu Z.H., Zhang J.J., Zhang C., Dong Y., Ren Z.Z., Gao Z., Liu M., Zhao H.J., Jia L. (2018). Antioxidative and hepatoprotective effects of enzymatic and acidic-hydrolysis of *Pleurotus geesteranus* mycelium polysaccharides on alcoholic liver diseases. Carbohydr. Polym..

[12] López-Legarda X., Rostro-Alanis M., Parra-Saldivar R., Villa-Pulgarín J.A., Segura-Sánchez F. (2021). Submerged cultivation, characterization and in vitro antitumor activity of polysaccharides from *Schizophyllum radiatum*. Int. J. Biol. Macromol..

[13] Moussa A.Y., Fayez S., Xiao H., Xu B. (2022). New insights into antimicrobial and antibiofilm effects of edible mushrooms. Food Res. Int..

[14] Krishnamoorthi R., Srinivash M., Mahalingam P.U., Malaikozhundan B. (2022). Dietary nutrients in edible mushroom, *Agaricus bisporus* and their radical scavenging, antibacterial, and antifungal effects. Process. Biochem..

[15] Song X.L., Ren Z.Z., Wang X.X., Jia L., Zhang C. (2020). Antioxidant, anti-inflammatory and renoprotective effects of acidic-hydrolytic polysaccharides by spent mushroom compost (*Lentinula edodes*) on LPS-induced kidney injury. Int. J. Biol. Macromol..

[16] Li J.H., Shi H., Li H., Luo Y.Y., Zhang M., Yu R.M., Huang W.J., Song L.Y., Zhu J.H. (2023). Structural elucidation and immunoregulatory activity of a new polysaccharide obtained from the edible part of *Scapharca subcrenata*. Process. Biochem..

[17] Jiao J.Q., Yong T.Q., Huang L.H., Chen S.D., Xiao C., Wu Q.P., Hu H., Xie Y., Li X., Liu Y. (2023). A *Ganoderma lucidum* polysaccharide F31 alleviates hyperglycemia through kidney protection and adipocyte apoptosis. Int. J. Biol. Macromol..

[18] Ge Y.Z., Qiu H.M., Zheng J.X. (2022). Physicochemical characteristics and anti-hyperlipidemic effect of polysaccharide from BaChu mushroom (*Helvella leucopus*). Food Chem. X.

[19] Wang X.Y., Yin J.Y., Nie S.P., Xie M.Y. (2018). Isolation, purification and physicochemical properties of polysaccharide from fruiting body of *Hericium erinaceus* and its effect on colonic health of mice. Int. J. Biol. Macromol..

[20] Chang C.J., Lin C.S., Lu C.C., Martel J., Ko Y.F., Ojcius D.M., Tseng S.F., Wu T.R., Chen Y.Y., Young J.D. (2015). *Ganoderma lucidum* reduces obesity in mice by modulating the composition of the gut microbiota. Nat. Commun..

[21] Liu Q., An X., Chen Y., Deng Y., Niu H., Ma R., Zhao H., Cao W., Wang X., Wang M. (2022). Effects of *Auricularia auricula* polysaccharides on gut microbiota and metabolic phenotype in mice. Foods.

[22] Li L., Guo W.L., Zhang W., Xu J.X., Qian M., Bai W.D., Zhang Y.Y., Rao P.F., Ni L., Lv X.C. (2019). *Grifola frondosa* polysaccharides ameliorate lipid metabolic disorders and gut microbiota dysbiosis in high-fat diet fed rats. Food Funct..

[23] Zhang C., Zhang L., Liu H., Zhang J.J., Hu C.L., Jia L. (2018). Antioxidation, anti-hyperglycaemia and renoprotective effects of extracellular polysaccharides from *Pleurotus eryngii* SI-04. Int. J. Biol. Macromol..

[24] Nakahara D., Nan C., Mori K., Hanayama M., Kikuchi H., Hirai S., Egashira Y. (2020). Effect of mushroom polysaccharides from *Pleurotus eryngii* on obesity and gut microbiota in mice fed a high-fat diet. Eur. J. Nutr..

[25] Wang L.Q., Xu N., Zhang J.J., Zhao H.J., Lin L., Jia S.H., Jia L. (2015). Antihyperlipidemic and hepatoprotective activities of residue polysaccharide from *Cordyceps militaris* SU-12. Carbohydr. Polym..

[26] Huang R., Zhu Z.J., Wu S.J., Wang J., Chen M.F., Liu W., Huang A.O., Zhang J.M., Wu Q.P., Ding Y. (2022). Polysaccharides from *Cordyceps militaris* prevent obesity in association with modulating gut microbiota and metabolites in high-fat diet-fed mice. Food Res. Int..

[27] Berger A., Rein D., Kratky E., Monnard I., Hajjaj H., Meirim I., Piguet-Welsch C., Hauser J., Mace K., Niederberger P. (2004). Cholesterol-lowering properties of *Ganoderma lucidum* in vitro, ex vivo, and in hamsters and minipigs. Lipids Health Dis..

[28] Feng T., Jia W., Wang W.H., Lin C.C., Fan H., Zhang J.S., Bao H.Y. (2016). Structural Characterization and Immunological Activities of a Novel Water-Soluble Polysaccharide from the Fruiting Bodies of Culinary-Medicinal Winter Mushroom, *Flammulina velutipes* (Agaricomycetes). Int. J. Med. Mushrooms.

[29] Jia W., Feng J., Zhang J.S., Lin C.C., Wang W.H., Chen H. (2017). Structural characteristics of the novel polysaccharide fvpa1 from winter culinary-medicinal mushroom, *Flammulina velutipes* (agaricomycetes), capable of enhancing natural killer cell activity against k562 tumor cells. Int. J. Med. Mushrooms.

[30] Wang W.H., Zhang J.S., Feng T., Deng J., Lin C.C., Fan H., Yu W., Bao H.Y., Jia W. (2018). Structural elucidation of a polysaccharide from *Flammulina velutipes* and its immunomodulation activities on mouse B lymphocytes. Sci. Rep..

[31] Jia F., Gao Y., Zhang J., Hou F., Shi J., Song S., Yang S. (2025). *Flammulina velutipes* mycorrhizae dietary fiber attenuates the development of obesity via regulating lipid metabolism in high-fat diet-induced obese mice. Front. Nutr..

[32] Masuko T., Minami A., Iwasaki N., Majima T., Nishimura S., Lee Y.C. (2005). Carbohydrate analysis by a phenol-sulfuric acid method in microplate format. Anal. Biochem..

[33] Smith P.K., Krohn R.I., Hermanson G.T., Mallia A.K., Gartner F.H., Provenzano M.D., Fujimoto E.K., Goeke N.M., Olson B.J., Klenk D.C. (1985). Measurement of protein using bicinchoninic acid. Anal. Biochem..

[34] Jia W., Wang W.H., Yu D.S., Yu Y.C., Feng Z., Li H.W., Zhang J.S., Zhang H.N. (2024). Structural elucidation of a polysaccharide from *Flammulina velutipes* and its lipid-lowering and immunomodulation activities. Polymers.

[35] Wei H., Yue S., Zhang S.Z., Lu L. (2018). Lipid-lowering effect of the *Pleurotus eryngii* (king oyster mushroom) polysaccharide from solid-state fermentation on both macrophage-derived foam cells and zebrafish models. Polymers.

[36] Barker S.A., Bourne E.J., Stacey M., Whiffen D.H. (1954). Infra-red spectra of carbohydrates. Part I. Some derivatives of d-glucopyranose. J. Chem. Soc..

[37] Staaf M., Urbina F., Weintraub A., Widmalm G. (1999). Structure elucidation of the o-antigenic polysaccharide from the enteroaggregative *Escherichia coli* strain 62d1. Eur. J. Biochem..

[38] Jansson P.E., Kenne L., Widmalm G. (1989). Computer-assisted structural analysis of polysaccharides with an extended version of casper using 1H- and 13C-N.M.R.data. Carbohydr. Res..

[39] Senchenkova S.N., Knirel Y.A., Shashkov A.S., Ahmed M., Mavridis A., Rudolph K. (2002). Structure of the o-polysaccharide of *Erwinia carotovora* ssp. *carotovora* gspb 436. Carbohydr. Res..

[40] Panchal S.K., Poudyal H., Arumugam T.V., Brown L. (2011). Rutin attenuates metabolic changes, nonalcoholic steatohepatitis, and cardiovascular remodeling in high-carbohydrate, high-fat diet-fed rats. J. Nutr..

[41] Katakami N., Kaneto H., Osonoi T., Saitou M., Takahara M., Sakamoto F., Yamamoto K., Yasuda T., Matsuoka T.A., Matsuhisa M. (2011). Usefulness of lipoprotein ratios in assessing carotid atherosclerosis in Japanese type 2 diabetic patients. Atherosclerosis.

[42] Katan M.B., Grundy S.M., Jones P., Law M., Miettinen T., Paoletti R., Stresa Workshop Participants (2003). Efficacy and safety of plant stanols and sterols in the management of blood cholesterol levels. Mayo Clin. Proc..

[43] Olofsson S.O., Borèn J. (2005). Apolipoprotein B: A clinically important apolipoprotein which assembles atherogenic lipoproteins and promotes the development of atherosclerosis. J. Intern. Med..

[44] Gotto A.M.J., Pownall H.J., Havel R.J. (1986). Introduction to the plasma lipoproteins. Methods Enzymol..

[45] Fielding C.J., Fielding P.E. (1995). Molecular physiology of reverse cholesterol transport. J. Lipid Res..

[46] Rader D.J. (2006). Molecular regulation of HDL metabolism and function: Implications for novel therapies. Nat. Rev. Drug Discov..

[47] Kuivenhoven J.A., Pritchard H. (2014). Reverse cholesterol transport: From classical view to new insights. Nat. Rev. Cardiol..

[48] Tanigawa H., Billheimer J.T., Tohyama J., Fuki I.V., Ng D.S., Rothblat G.H., Rader D.J. (2009). Lecithin: Cholesterol acyltransferase expression has minimal effects on macrophage reverse cholesterol transport in vivo. Circulation.

[49] Chang S.S., Lei X., Xie Q., Zhang M.J., Zhang Y.G., Xi J.X., Duan J.Y., Ge J., Nian F.Z. (2024). In vitro study on antioxidant and lipid-lowering activities of tobacco polysaccharides. Bioresour. Bioprocess..

[50] Cheng F.E., Yang Y.R., Yun S.J., Cao J.L., Chang M.C., Cheng Y.F., Feng C.P. (2023). *Sparassis latifolia* Polysaccharide Attenuates Cholesterol in Rats Maintained on a High-Fat, High-Cholesterol Diet. J. Food Biochem..

[51] Filipowicz W., Bhattacharyya S.N., Sonenberg N. (2008). Mechanisms of post-transcriptional regulation by microRNAs: Are the answers in sight?. Nat. Rev. Genet..

[52] Watanabe M., Houten S.M., Mataki C., Christoffolete M.A., Kim B.W., Sato H., Messaddeq N., Harney J.W., Ezaki O., Kodama T. (2006). Bile acids induce energy expenditure by promoting intracellular thyroid hormone activation. Nature.

[53] Wang Y.D., Chen W.D., Moore D.D., Huang W. (2008). FXR: A metabolic regulator and cell protector. Cell Res..

[54] Staley C., Weingarden A.R., Khoruts A., Sadowsky M.J. (2017). Interaction of gut microbiota with bile acid metabolism and its influence on disease states. Appl. Microbiol. Biotechnol..

[55] Hu R.K., Guo W.L., Huang Z.R., Li L., Liu B., Lv X.C. (2018). Extracts of *Ganoderma lucidum* attenuate lipid metabolism and modulate gut microbiota in high-fat diet fed rats. J. Funct. Foods.

[56] Xu X.F., Zhang X.W. (2015). *Lentinula edodes*-Derived Polysaccharide Alters the Spatial Structure of Gut Microbiota in Mice. PLoS ONE.

[57] Ottman N., Geerlings S.Y., Aalvink S., de Vos W.M., Belzer C. (2017). Action and function of *Akkermansia muciniphila* in microbiome ecology, health and disease. Best Pract. Res. Clin. Gastroenterol..

[58] Zhou K. (2017). Strategies to promote abundance of *Akkermansia muciniphila*, an emerging probiotic in the gut, evidence from dietary intervention studies. J. Funct. Foods.

[59] Chen Y.T., Yang N.S., Lin Y.C., Ho S.T., Li K.Y., Lin J.S., Liu J.R., Chen M.J. (2018). A combination of *Lactobacillus mali* APS1 and dieting improved the efficacy of obesity treatment via manipulating gut microbiome in mice. Sci. Rep..

[60] An H.M., Park S.Y., Lee D.K., Kim J.R., Cha M.K., Lee S.W., Lim H.T., Kim K.J., Ha N.J. (2011). Antiobesity and lipid-lowering effects of *Bifidobacterium* spp. in high fat diet-induced obese rats. Lipids Health Dis..

[61] Krumbeck J.A., Rasmussen H.E., Hutkins R.W., Clarke J., Shawron K., Keshavarzian A., Walter J. (2018). Probiotic Bifidobacterium strains and galactooligosaccharides improve intestinal barrier function in obese adults but show no synergism when used together as synbiotics. Microbiome.

[62] Grossi E., Pace F. (2016). The Gut Microbiota and Obesity in Humans.

